# Egyptian patients with cleft lip: our experience with primary rhinoplasty

**DOI:** 10.1186/s40902-024-00448-3

**Published:** 2024-11-04

**Authors:** Adel Mabrouk, Mohamed Samir Badawy, Mai Raafat Hammad, Amr Mabrouk

**Affiliations:** Department of Plastic, Burn and Maxillofacial Surgery, Ain Shams University, Egy54 Abdullah Ebn Taher Street, Nasr City, Cairo, 11731 Egypt

## Abstract

**Background:**

Controversy exists regarding the timing of rhinoplasty for patients with cleft lip as some surgeons shy away from primary correction for fear of causing harm to the growth of the nose and the maxilla.

We present our favorable experience with primary rhinoplasty with repair of unilateral cleft lip in Ain Shams University plastic surgery department, providing insights into the specific management of patients of middle eastern descent.

**Methods:**

Prospective study of 32 patients, ages 3 months- 1 year with unilateral CL presenting to Ain Shams University hospitals between January 2019 and July 2022. Primary rhinoplasty was performed at the time of lip repair. Lip repair was done by Tennison-Randall technique. Evaluation of results was done by expert analysis of photographs, anthropometric measurements, and parents’ satisfaction.

**Results:**

Over-all concordance rate was 93% and inter-observer concordance was 89%. Non-significant differences were found between cleft and noncleft sides regarding nostril dome height, columellar length, and alar width 6 months post-operatively. 81.25% of the parents were very satisfied with the results.

**Conclusion:**

Our study targeted a cohort of Egyptian patients with unilateral cleft lip, who underwent primary rhinoplasty and the time of lip repair, showing favorable results, supporting the literature advocating for this timing, but limited by relatively short follow up period. To the best of the authors’ knowledge, this is the first study in Egypt to highlight the outcomes and direct experience for primary rhinoplasty with cleft lip repair in an Egyptian population.

**Supplementary Information:**

The online version contains supplementary material available at 10.1186/s40902-024-00448-3.

## Background

Controversy exists regarding the timing of rhinoplasty for patients with cleft lip (CL) [1]. Timing can be divided into primary, intermediate, and definitive. Primary rhinoplasty is performed at the time of CL repair, around 3–6 months of age. The reason for controversy regarding primary rhinoplasty is based on the potential interference with midface growth due to manipulation of nasal cartilage, which is originally based on experimental studies [2, 3]. However, multiple studies have demonstrated that repositioning of lower lateral cartilage (LLC) without cartilage resection did not interfere with subsequent nasal and midfacial development [4–8] Primary rhinoplasty allows for early restoration of shape and potential for symmetric nasal growth as the child grows and helps to avoid psychological impact during formative years [9, 10]. It closes the nasal floor, optimizes nasal tip support, eliminates asymmetry of the alar base [6] and requires less complex secondary procedure [6, 11].

Intermediate rhinoplasty is performed before school age, usually between 4–6 years of age. Definitive rhinoplasty is performed after skeletal maturity, but relies on a stable maxillary skeletal foundation, which may require orthodontics, alveolar bone grafting, and sometimes orthognathic surgery [1].

The appropriate technique and timing are still debatable in the literature [1].

In unilateral cleft lip, cleft side has a shorter columella, elongated lateral crus of LLC, flattened ala with horizontal orientation of the nostril, nasal floor that is caudal and absent, and an alar base that is laterally and posteriorly displaced. There is deviation of the columella to the non-cleft side, with deviation of septum and anterior nasal spine to the non-cleft side [12].

To the best of our knowledge, this is the first study to highlight the outcomes and direct experience for primary rhinoplasty with cleft lip repair in an Egyptian population.

The aim of this study is to present our favorable experience with primary rhinoplasty in our institution’s plastic surgery department, which may provide insights into the specific management of patients of middle eastern descent with CL.

## Methods

Prospective study of 32 patients with unilateral CL presenting to Ain Shams University hospitals between January 2019 and July 2022.

Included in the study were patients ages 3 months—1 year of age of both genders with unilateral CL.

In all patients, primary rhinoplasty was performed at the time of lip repair. Lip repair was done by Tennison-Randall technique [13, 14]. Rhinoplasty was performed by dissection of the cleft side LLC and alar cartilage in the supraperichondrial plane to completely free it from the skin, nasal mucosa and abnormal attachments, through the lip repair incisions, plus extended columellar incision and alar rim incisions if needed, to allow for cartilage manipulation and skin redraping, then multiple suspension sutures were used with PDS 5–0 Ethicon® to reposition the LLC, and suspend it, allowing shifting of the alar dome at the genu, starting the sutures at the nasal mucosa, through the cartilage, then tying the sutures over a bolster in the direction of the nostrils. (Video 1). A nasal retainer was inserted after skin closure, and maintained for 3 months.

**Fig. 1:**
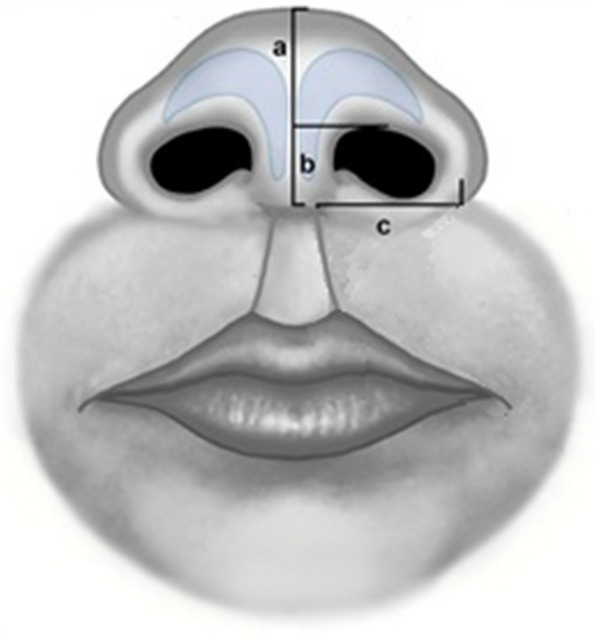
Anthropometric measurements of nasal dimensions. **a** Nostril dome height (**b**) Columellar length (**c**) Alar width

Evaluation of results was done by photographic assessment, anthropometric measurements, and parents’ satisfaction. All patients underwent the following measurements bilaterally to compare cleft side to normal side [15], 6 months post-operatively, as shown in Fig. 1:

- Nostril dome height: Measurement from the lateral border at the base of the columella to the highest point on the nasal dome.
- Columellar length: Measurement from the lateral border of the base of the columella to the highest point of the nostril at the same level.
- Alar width: Measured from the lateral border at the base of the columella to the most lateral point of the ala in a line perpendicular to the axis of the columella.

These measurements were selected because they reflect the symmetry and centralization of nasal tip, the correction of columella to match non-cleft side and the correction of widened ala and horizontal nostril shape to match the non-cleft side [16]. Measurements were performed by two separate operators.

Parents’ satisfaction was assessed through a 5-point Likert Scale regarding their satisfaction with the cosmetic appearance of the repaired nasal deformity post-operatively, in which 1 meant very dissatisfied, 2 meant dissatisfied, 3 meant neutral, 4 meant satisfied and 5 meant very satisfied.

Statistical analysis was done by SPSS 16.0 statistical software package. Results are presented in the form of percentage for qualitative data, mean and standard deviation in case of quantitative variables. Statistical analysis for pre- and postoperative findings was performed using Mann–Whitney U Test.

The results were considered statistically significant if *P* value was below 0.05.

## Results

**Fig. 2:**
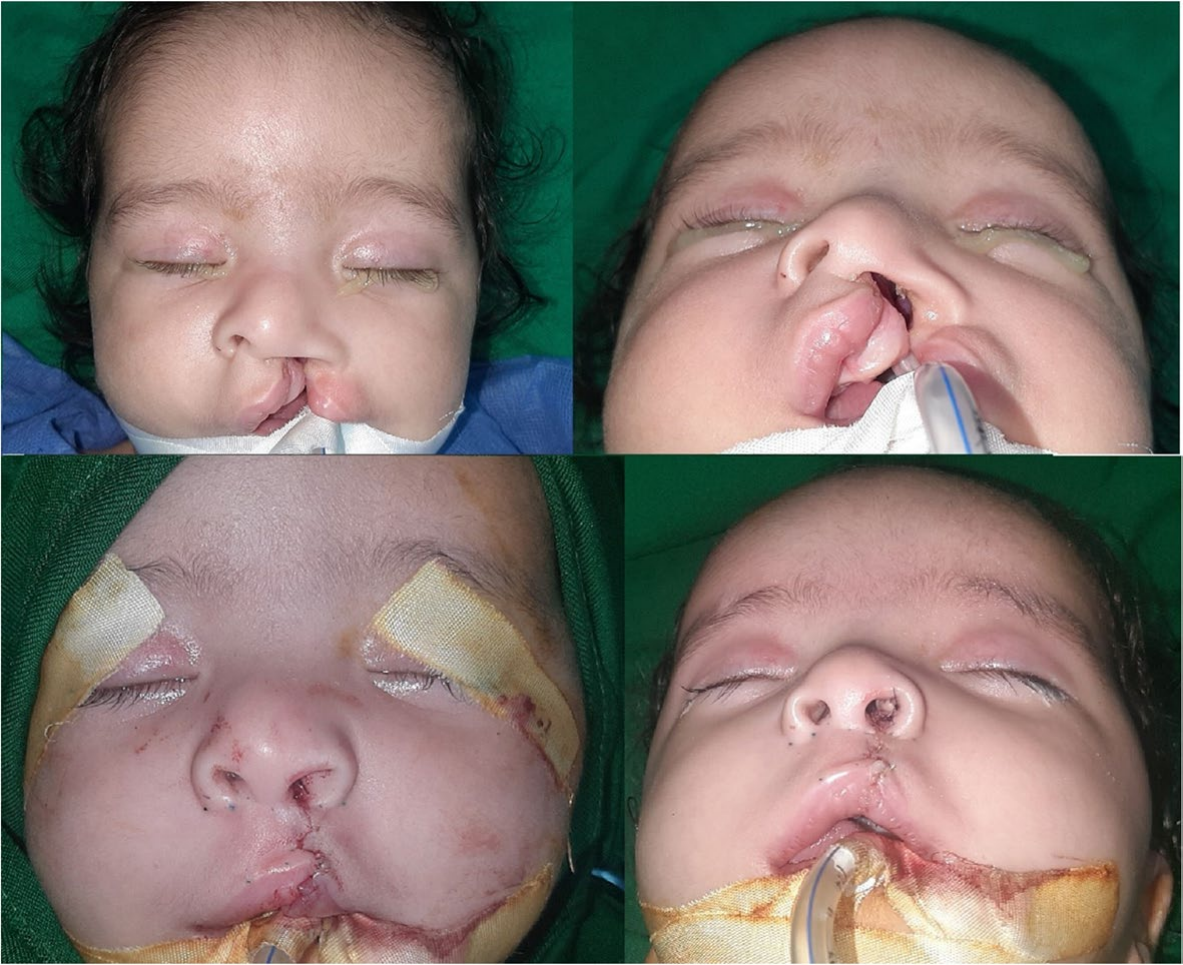
5-month-old patient with unilateral cleft lip. **A** Pre-operative photos. **B** Post-operative photos

(A) Photographic assessment:

Pre-operative and post-operative photographs were analyzed by three plastic surgery consultants not involved in the study regarding symmetry and correction of cleft side deformities. The over-all concordance rate was 93% and inter-observer concordance was 89%, demonstrating subjectively good results, with high agreement among the consultants. Figure 2 provides pre-operative and post-operative photos of a 5-month-old with left unilateral cleft lip, which shows correction of columellar deviation to the midline, correction of columellar length on cleft side by shortening the lateral crus of LLC and recruiting it to lengthen the columella, nostrils with better symmetry and a more vertical orientation, correction of alar base position to a more medial and cranial position, and repair of nasal floor.

(B) Anthropometric measurements:

Non-significant differences were found between cleft and noncleft sides regarding nostril dome height, columellar length, and alar width 6 months post-operatively (Table 1).

**Table 1:** Mean and standard deviation for anthropometry

Measurements (millimeters)	Cleft side Mean (Standard deviation)	Noncleft side Mean (Standard deviation)	*P* value
Nostril dome height	9.81 (1.09)	10 (3.3)	0.525
Columellar length	4.48 (0.82)	4.64 (0.67)	0.432
Alar width	13.89 (1.17)	13.75 (1.07)	0.634

(C) Parents’ satisfaction:

81.25% of the parents were very satisfied with the results, and 18.75% were satisfied.

## Discussion

Nasal deformities are inseparable from lip deformity in patients with CL and should be tackled at the same time of surgery to minimize further deformity in the nasolabial region and the need for subsequent interventions.

Cleft nasal deformities noted with unilateral CL include deviation of columella towards noncleft side, wider and retro-placed nostril on cleft side, postero-laterally displaced alar base on cleft side, postero-laterally displaced cleft side dome of LLC, increased angle between medial and lateral crura on cleft side with short medial crus on cleft side and long lateral crus on cleft side [17].

Despite the controversy regarding timing of nasal repair [1], over fifty percent of cleft surgeons prefer to do some form of primary rhinoplasty in infancy [18], with rising trends [19]. The rationale is to make use of the malleability of the nasal cartilages and soft tissues [20] which reduces the severity of the nasal deformity, reduces the number of required revision surgeries upon skeletal maturity. Children with orofacial cleft suffer the highest incidence of depression, anxiety, and negative peer-relationships around the ages 8 to 10 years [21]. This supports the need for early intervention before social integration to avoid negative psychosocial experiences [11, 21].

Historically, cleft surgeons were influenced by animal studies that suggested that rhinoplasty during skeletal growth could induce long-term nasal growth disruption due to affection of growth centers [2, 3]. However, rigorous evidence is lacking pertaining to disruption, with multiple long-term studies disproving it [4–8].

Our study targeted a cohort of Egyptian infants and agrees with the literature advocating for primary rhinoplasty in cleft lip patients. Primary rhinoplasty in our patients allowed for correction of columellar deviation, columellar length, nostril asymmetry and orientation, and alar base position with reliable results after 6 months. Cleft and non-cleft sides showed non-significant differences regarding nostril dome height, columellar length, and alar width 6 months post-operatively.

Further research is needed into the specific challenges regarding this population over the long term.

## Conclusions

Our study targeted a cohort of Egyptian infants, agreeing with the literature advocating for primary rhinoplasty in cleft lip patients using anthropometry, photographic analysis and parent satisfaction.

## Supplementary Information

Supplementary Material 1: Video 1. Dissection and suspension of lower lateral cartilage.

## Data Availability

All data and material supporting the findings of this study is available within the manuscript and supplementary materials. Additional data can be provided upon request.

## Abbreviations

CL: Cleft lip
LLC: Lower lateral cartilage
